# Self-reported adverse events after 2 doses of COVID-19 vaccine in Korea

**DOI:** 10.4178/epih.e2023006

**Published:** 2022-12-26

**Authors:** Yunhyung Kwon, Insob Hwang, Mijeong Ko, Hyungjun Kim, Seontae Kim, Soon-Young Seo, Enhi Cho, Yeon-Kyeng Lee

**Affiliations:** 1 Adverse Event Information Analysis Team, Immunization Safety Group, COVID-19 Vaccination Task Force, Korea Disease Control and Prevention Agency, Cheongju, Korea; 2 Adverse Event Management Team, Immunization Safety Group, COVID-19 Vaccination Task Force, Korea Disease Control and Prevention Agency, Cheongju, Korea; 3 Immunization Safety Group, COVID-19 Vaccination Task Force, Korea Disease Control and Prevention Agency, Cheongju, Korea

**Keywords:** Safety, COVID-19 vaccine, Heterologous, Homologous, Prime-boost

## Abstract

**OBJECTIVES:**

In Korea, a national coronavirus disease 2019 (COVID-19) vaccination program was implemented, including 4 vaccines against COVID-19. A text messaging-based survey, in addition to a passive adverse event reporting system, was launched to quickly report unusual symptoms post-vaccination. This study compared the frequency of adverse events after COVID-19 vaccination based on the vaccine type and the type of 2-dose regimen (homologous or heterologous).

**METHODS:**

Self-reported adverse events were collected through a text-message survey for 7 days after each vaccination. This study included 50,950 vaccine recipients who responded to the survey at least once. Informed consent to receive surveys via text was obtained from the vaccine recipients on the date of first vaccination.

**RESULTS:**

The recipients of mRNA vaccines reported local and systemic reactions 1.6 times to 2.8 times more frequently after dose 2 than after dose 1 (p<0.001), whereas ChAdOx1-S recipients reported significantly fewer local and systemic reactions after dose 2 than after dose 1 (p<0.001). Local and systemic reactions were approximately 2 times and 4 times more frequent for heterologous vaccination than for BNT162b2/BNT162b2 and ChAdOx1-S/ChAdOx1-S regimens, respectively. Young individuals, female, and those receiving heterologous vaccine regimens including ChAdOx1-S/BNT162b2 vaccines reported more adverse events than older participants, male, and those with homologous vaccine regimens.

**CONCLUSIONS:**

Although a heterologous regimen, youth, and female sex were associated with a higher risk of adverse reactions after COVID-19 vaccination, no critical issues were noted. Active consideration of heterologous schedules based on the evidence of efficacy and safety appears desirable.

## GRAPHICAL ABSTRACT

[Fig f2-epih-45-e2023006]

## INTRODUCTION

The World Health Organization declared coronavirus disease 2019 (COVID-19), which is caused by severe acute respiratory syndrome coronavirus 2 (SARS-CoV-2), a public health emergency of international concern on January 30, 2020 and a pandemic shortly afterward [1]. Vaccination is a key measure for reducing the severity of COVID-19 and the incidence of hospitalization. In the vaccination campaign in Korea, 4 COVID-19 vaccines were in use as of December 2021; the rollout of the AstraZeneca vaccine (ChAdOx1-S, hereafter ChAd) and the Pfizer-BioNTech vaccine (BNT162b2, hereafter BNT) began in February 2021, while the rollout of the Janssen vaccine (Ad26.COV2.S, hereafter Ad26) and the Moderna vaccine (mRNA-1273) began in June 2021 [2-5]. Although the Korean government had contracted for enough COVID-19 vaccines to cover the population, vaccine supplies were limited and were delivered to Korea gradually in the first 2 quarters of 2021. The roadmap for the allocation and prioritization of COVID-19 vaccinations was designed with the help of the Korea Advisory Committee on Immunisation Practices (KACIP) for maximum public health impact, considering vaccine availability and the epidemiologic setting. The group at highest priority for vaccination included residents and staff at long-term care facilities, older adults, and people in frequent close contact with COVID-19 patients. The recommendations were expanded to include people serving in critical societal functions, such as soldiers and police officers [4-6].

A primary series of 2 doses separated by an 8-week to 12-week interval (12 weeks for ChAd and 8 weeks for BNT) was recommended for all of the vaccines except Ad26, which was administered as a single dose [7]. Each primary series was recommended to include the same vaccine for both doses (a homologous regimen); however, the KACIP revised these guidelines to recommend that ChAd recipients receive BNT as their second dose (constituting a heterologous regimen) given safety issues.

As each brand of COVID-19 vaccine was introduced, to monitor vaccine safety, the Korea Disease Control and Prevention Agency (KDCA) conducted active text message–based monitoring of the first 10,000 people who received each vaccine. This resembled the method used with the v-safe system, a web-based questionnaire for vaccine recipients in the United States [8], and the VAC4EU initiative, part of the Early-COVID-Vaccine-Monitor project in the European Union [9]. Health professionals must quickly collect information about adverse events (AEs) during early stages of vaccination efforts and inform the public about real-world data; additionally, safety concerns are particularly important because vaccines are designed for disease prevention, not treatment. For this reason, and given the limited patient experience, the KDCA performed close monitoring as the COVID-19 vaccine of each brand was rolled out. Increased monitoring of AEs was particularly crucial for the heterologous regimen because Korea was recommending it for the first time, although such a regimen had already been used in other countries. This study was designed to describe and compare common AEs following immunisation (AEFIs) based on self-reports from participants who received either homologous or heterologous 2-dose regimens of the 4 aforementioned COVID-19 vaccines.

## MATERIALS AND METHODS

As each brand of COVID-19 vaccine was rolled out, the KDCA conducted a text messaging–based survey regarding AEs among vaccine recipients for 7 consecutive days beginning on the date of COVID-19 vaccination. Text-message questions about AEs were sent to vaccine recipients until the number of respondents reached approximately 10,000 for each brand of COVID-19 vaccine. Only people who responded at least once to the text survey during the 7 consecutive days after the first vaccination were included in this study and received the text-based questionnaire after their second vaccination. Informed consent to receive surveys via text was obtained from the vaccine recipients on the day of the first vaccination.

### Study population

A total of 50,950 individuals were surveyed: 12,854 ChAd recipients vaccinated between February 26 and March 8, 2021; 10,292 BNT recipients vaccinated between February 27 and April 3, 2021; 7,567 mRNA-1273 recipients vaccinated between June 24 and June 30, 2021; 10,220 Ad26 recipients vaccinated on June 10, 2021; and 10,017 BNT recipients vaccinated between July 8 and July 9, 2021 who had received ChAd as their first dose.

The survey response rates after the first vaccination by vaccine brand were as follows: 25.8% (12,854 of 49,803 people) for ChAd, 22.8% (10,292 of 45,090 people) for BNT, 46.7% (7,567 of 16,205 people) for mRNA-1273, and 20.4% (10,220 of 49,995 people) for Ad26. In addition, the response rate for a heterologous regimen after a second vaccination with BNT was 19.6% (10,017 of 51,032 people).

### Questionnaires

The structured questionnaire enquired about frequently reported AEFIs, medical services or medication sought, sex, and age group. AEFIs were divided into local reactions, systemic reactions, and limitations on activities of daily living due to COVID-19 vaccination. The local reactions included pain, swelling, redness, itching, and urticaria at the injection site, while the systemic reactions included fever, chills, headache, arthralgia, myalgia, fatigue, nausea, vomiting, diarrhoea, abdominal pain, rash, and armpit pain. The questionnaire also asked about any limitations in daily life activities due to symptoms and whether medical help was sought.

### Age group categorisation

Recipients were categorised into 5 groups based on age: ≤29 years, 30-39 years, 40-49 years, 50-59 years, and ≥ 60 years. Although the target age group differed for each vaccine, analysis was performed without adjustment. Those who reported symptoms at least once during days 0 to 7 were considered symptomatic.

### Statistical analysis

We performed descriptive analysis for the reported AEs and cross-analysis or Fisher exact testing to compare the proportion of respondents by vaccine brand and dose. Results are presented as frequencies, percentages, standard errors, and means with standard deviations. Multivariate logistic regression analysis was applied with sex, age, and vaccine type as explanatory variables to identify risk factors for adverse reactions by administered dose. Odds ratios (ORs) and 95% confidence intervals (CIs) were presented. A p-value of < 0.05 was considered to indicate statistical significance. SAS version 9.4 (SAS Institute Inc., Cary, NC, USA) was used for all analyses.

### Ethics statement

Given that the current activity was conducted and authorised by the public health authority and the purpose was to disseminate information to the public, the current study was exempted from the requirement to obtain consent to participate by ethical board review (No. 2022-0349-001, Public Institutional Review Board Designated by Ministry of Health and Welfare).

## RESULTS

### Distribution of respondents by sex, age, and COVID-19 vaccine type

The study group comprised 50,950 respondents, all aged ≥18 years, who received a primary series of COVID-19 vaccines. Female accounted for 70-80% of responses for each vaccine type except Ad26, for which male accounted for 95.9% of responses. For ChAd, the highest proportion of respondents after the first vaccination came from the 50-year to 59 year age group (26.2%) and the highest proportion after the second vaccination from the 40 year to 49 year group (25.7%). The smallest proportion of respondents was those at least 60 years old, at 7.5% of total ChAd respondents after the first vaccination and 7.1% after the second vaccination; otherwise, all age groups had similar response rates. For BNT, the largest proportion of respondents were those aged 29 years or younger, constituting 30.7% of those responding after the first dose and 30.3% of respondents after the second dose. The proportion of respondents also tended to decrease with increasing age; however, for those aged ≥ 60 years, the first-dose and second-dose response proportions of 12.9% and 11.5%, respectively, resembled those among participants aged 50 years to 59 years. For mRNA-1273, most respondents were ≤ 29 years old (90.5% in the first round, 89.8% in the second dose). For Ad26, the majority of respondents (83.2%) were from the 30-year to 39 year age group. In the heterologous ChAd/BNT group, 33.5% of respondents were aged 50 years to 59 years. The intervals between doses were 79.4 ± 3.9 days for ChAd/ChAd, 77.5± 3.9 days for ChAd/BNT, 28.6± 2.5 days for mRNA-1273/mRNA-1273, and 21.1 ± 1.5 days for BNT/BNT (Table 1).

### Safety profile after COVID-19 vaccination

**Figure 1.**
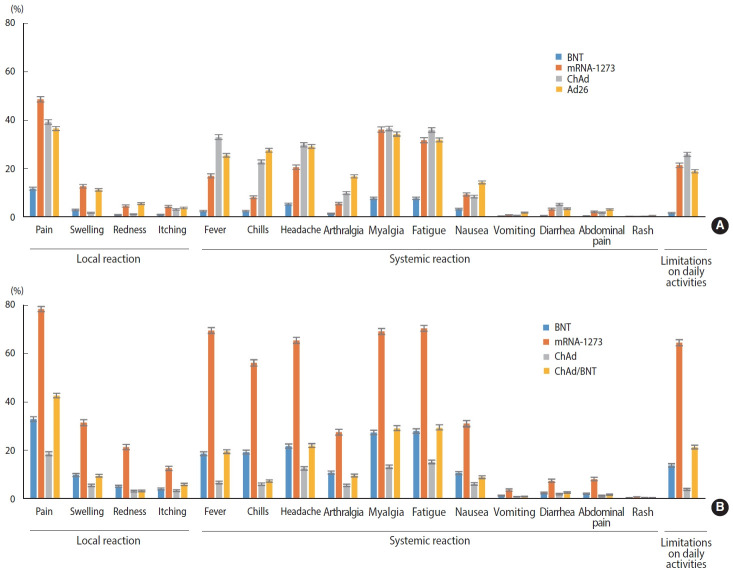
Solicited adverse reactions after the first dose (A) and the second dose (B) of COVID-19 vaccine. COVID-19, coronavirus disease 2019; ChAd, AstraZeneca COVID-19 vaccine (ChAdOx1-S); BNT, Pfizer-BioNTech COVID-19 vaccine (BNT162b2); Ad26, Janssen COVID-19 vaccine (Ad26.COV2.S); mRNA-1273, Moderna COVID-19 vaccine (messenger RNA-1273).

Generally, recipients of mRNA-based vaccines reported 1.6-2.8 times more frequent local and systemic reactions after the second dose than after the first dose (p< 0.001), whereas ChAd recipients reported significantly fewer local and systemic reactions after the second dose than after the first dose (p< 0.001). After the first dose, mRNA-1273 recipients reported significantly higher rates of both local (approximately 4 times greater) and systemic (approximately 2.4 times greater) reactions than BNT recipients (p< 0.001). For the second dose, mRNA-1273 recipients reported 3.6 times and 2.3 times higher rates of local and systemic reactions, respectively, than BNT recipients, and heterologous regimen (ChAd/ BNT) recipients exhibited approximately 1.3 times higher rates of local and systemic reactions than BNT/BNT recipients (p< 0.001). Ad26 recipients reported a slightly higher rate of local and systemic reactions than the recipients of ChAd dose 1 (p< 0.001). Pain and swelling were the most common local reactions regardless of the vaccine type and dose. Among systemic reactions, respondents reported myalgia and fatigue at high rates; the incidence of myalgia was reportedly higher after the first dose than the second, while that of fatigue was higher after the second dose. Regarding limitations in activities of daily living after vaccination, the highest observed rate was 64.7%, reported after the second dose of mRNA-1273 (Figure 1 and Table 2).

### Factors influencing the risk of adverse events after COVID-19 vaccination

Younger age, relative to participants ≥ 60 years old, was associated with a higher OR of reporting any AE or limitation in activities of daily living after any type of COVID-19 vaccine and dose (p< 0.05). Female had over 1.6 times greater risk of developing AEs or limitations in activities of daily living than male (p< 0.05). Among recipients of a first dose of COVID-19 vaccine, the recipients of mRNA-1273 and viral vector-based vaccines showed a higher prevalence of AEs and limitations in activities of daily living compared to the recipients of BNT. After the second dose, relative to homologous BNT, homologous ChAd appeared to be associated with lower rates of AEs and limitations in activities of daily living. Among recipients of heterologous COVID-19 vaccines, ChAd/BNT recipients had approximately 2 times greater odds of reporting AEs and limitations in activities of daily living compared with homologous BNT/BNT recipients. The homologous ChAd/ChAd regimen was associated with lower risk of reporting local symptoms (OR, 0.49; 95% CI, 0.46 to 0.53), systemic symptoms (OR, 0.54; 95% CI, 0.51 to 0.58), and limitations in activities of daily living (OR, 0.25; 95% CI, 0.22 to 0.29) (Table 3).

## DISCUSSION

As of December 2021, 2 mRNA-based vaccines (BNT and mRNA-1273) and 2 vector-based vaccines (ChAd and Ad26) were in use in Korea. Based on a previous report, after approximately 83.0% of those at least 12 years old in Korea had completed a primary series of COVID-19 vaccinations, 96.4% of the AEFIs reported to the Korea COVID-19 vaccine management system were non-serious [10]. Similar to v-safe, the smartphone-based survey system used in the United States [11], the KDCA carried out a text-message survey to quickly and easily capture unusual symptoms after COVID-19 vaccination. Among respondents, those who received mRNA-based COVID-19 vaccines reported local and systemic AEs more frequently after the second dose than the first. Among respondents administered ChAd, solicited local and systemic AEs were far less frequent after the second dose than the first. In a similar study in India, among people who received the Covishield (ChAdOx1-S [non-replicating viral vector]) vaccine—a commercial name for the AstraZeneca COVID-19 vaccine manufactured by Serum Institute of India Pvt Ltd.—the AE reporting rate decreased after the second dose (57.0 vs. 14.1%) [12]. Regarding the mRNA vaccines, the response rate of BNT recipients regarding all reactions was significantly lower than that among the mRNA-1273 recipients. Recipients of BNT reported lower rates than the incidence rates of various adverse reactions described in the approval of the Ministry of Food and Drug Safety [2]. This may be reflected in the preference for BNT in Korea [7], while higher rates reported among mRNA-1273 and Ad26 recipients might be due to the age difference among recipients, as these 2 vaccines were mostly administered to people 20 years to 30 years old. Some studies have shown that female and younger age groups had greater risk of AEs [13-16], perhaps related to the differing immune responses to antigens among different sex and age groups [17,18]. In contrast, at the early stage of vaccination with each vaccine brand, people may have been particularly sensitive to AEs, as indicated by the AE reporting rates from the national passive surveillance system dropping from 1.8% in the first week of vaccination (the first week of March) to 0.29% in the 12th week in May, then increasing to 0.71% in the 16th week in June 2021, when the rollout of mRNA-1273 and Ad26 began [19]. Moreover, vaccine recipients reported active concern regarding severe AEs, such as thrombosis with thrombocytopenia syndrome (TTS). In Korea, the first TTS case was reported on May 31, 2021 after a viral vector-based vaccine rollout, following the European Medicines Agency review published in April 2021 of an unusual blood clot after ChAd vaccination. Safety concerns resulted in the revision of the recommended ages to at least 50 years old for the ChAd vaccine and at least 30 years old for Ad26 (previously, those vaccines were recommended to those 18 years old and over).

Heterologous vaccine regimens against COVID-19 have been discussed as a means to improve the immunogenicity and safety of COVID-19 vaccines based on the risk of TTS due to ChAd, which was reported in Korea as well as elsewhere [20,21]. The KACIP recommended mRNA-based vaccines for people aged under 50 years as their second dose after initial immunisation with ChAd soon after the World Health Organization announced a risk of TTS in people aged ≤ 50 years [22]. Some countries have recommended heterologous regimens to deal with shortages in vaccine supply as well as to enhance clinical efficacy [23,24]. In Germany, heterologous (ChAd/BNT) immunisation with a 10-week to 12-week interval was recommended due to being well-tolerated and presenting improved immunogenicity compared with the homologous immunisation regimen [21]. Moreover, different platform vaccines are recommended for people who experienced anaphylaxis from their previous dose [25]. Some studies have shown a more robust immune response and higher reactogenicity in those receiving a heterologous ChAd/BNT regimen than in those vaccinated with ChAd/ChAd or BNT/BNT [21,26-28]. Our finding is consistent with those of other studies reporting increased AEs after heterologous prime-boost schedules. Furthermore, the reactogenicity data in the Oxford Vaccine Group-led Com-COV trial presented clearly increased reactogenicity after a heterologous boost with BNT after initial vaccination with ChAd [29]. Although heterologous schedules have been discussed in the context of efficacy and safety concerns, no critical issues related to those schedules have arisen; rather, they have helped address the poor or unstable supply of vaccines and achieve higher COVID-19 vaccination rates. Active consideration of heterologous schedules based on the evidence of efficacy and safety appears desirable.

This study has some limitations. First, these symptoms are based on self-reported responses and were not evaluated or medically diagnosed. According to the Korean national COVID-19 vaccination policy, target groups were assigned for each COVID-19 vaccine, and the demographic characteristics for each vaccine were not homogeneously distributed or comparable with the population profile. Additionally, the recipients of the text-based survey were particularly likely to be cautious about health matters because the survey was implemented in the early stages of each vaccine rollout. We also relied on participants reporting symptoms after vaccination. However, these potential biases should have been equivalent across the different schedules. Our study provides important real-world evidence for the safety and reactogenicity of the homologous and heterologous regimens. The results indicated that young people, female, and recipients of a heterologous regimen had particularly high risk of solicited local and systemic reactions after COVID-19 vaccination. Further studies on the mechanism of immunogenicity and reactogenicity of the COVID-19 vaccine, including long-term safety, are needed.

## Figures and Tables

**Figure f2-epih-45-e2023006:**
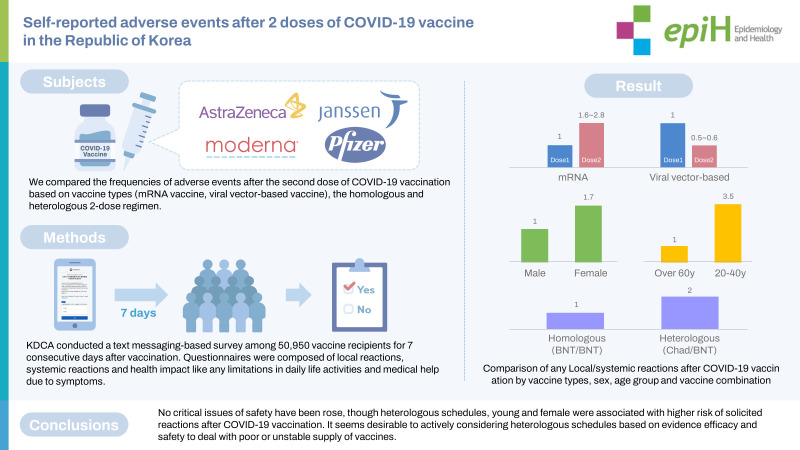


**Table 1.** Characteristics of respondents by coronavirus disease 2019 (COVID-19) vaccine type and dose (n=50,950)

Characteristics		ChAd		BNT		mRNA-1273		Ad26	Heterologous (ChAd/BNT)^1^
		Dose 1 (n=12,854)	Dose 2 (n=8,563)	Dose 1 (n=10,292)	Dose 2 (n=8,775)	Dose 1 (n=7,567)	Dose 2 (n=5,500)	Dose 1 (n=10,220)	Dose 2 (n=10,017)
Sex									
	Male	2,719 (21.2)	1,837 (21.5)	2,841 (27.6)	2,413 (27.5)	1,764 (23.3)	1,183 (21.5)	9,799 (95.9)	1,848 (18.4)
	Female	10,135 (78.8)	6,726 (78.5)	7,451 (72.4)	6,362 (72.5)	5,803 (76.7)	4,317 (78.5)	421 (4.1)	8,169 (81.6)
Age (yr)									
	≤29	2,647 (20.6)	1,701 (19.9)	3,158 (30.7)	2,656 (30.3)	6,850 (90.5)	4,938 (89.8)	0 (0.0)	2 (0.0)
	30-39	2,756 (21.4)	1,900 (22.2)	2,778 (27.0)	2,443 (27.8)	515 (6.8)	387 (7.0)	8,503 (83.2)	1,283 (12.8)
	40-49	3,112 (24.2)	2,204 (25.7)	1,805 (17.5)	1,600 (18.2)	144 (1.9)	125 (2.3)	1,527 (14.9)	2,652 (26.5)
	50-59	3,369 (26.2)	2,151 (25.1)	1,224 (11.9)	1,065 (12.1)	52 (0.7)	46 (0.8)	160 (1.6)	3,351 (33.5)
	≥60	970 (7.5)	607 (7.1)	1,327 (12.9)	1,011 (11.5)	6 (0.1)	4 (0.1)	30 (0.3)	2,729 (27.2)
Interval (day)^2^									
	Mean±SD	79.4±3.9		21.1±1.5		28.6±2.5			77.5±3.9
	Range	63-126		17-98		24-75			28-128

Values are presented as number (%). SD, standard deviation; ChAd, AstraZeneca COVID-19 vaccine (ChAdOx1-S); BNT, Pfizer-BioNTech COVID-19 vaccine (BNT162b2); Ad26, Janssen COVID-19 vaccine (Ad26.COV2.S); mRNA-1273, Moderna COVID-19 vaccine (messenger RNA-1273).

1 Received ChAd for the first dose and BNT for the second dose.

2 Interval between doses.

**Table 2.** Comparison of adverse reactions and health status after the first and second doses of coronavirus disease 2019 (COVID-19) vaccines

Variables		mRNA-based vaccine (%)			p-value^2^	Viral vector-based vaccine (%)		p-value^2^
		BNT	mRNA-1273	Heterologous^1^ (ChAd/BNT)		ChAd	Ad26	
Any local reaction								
	Dose 1	12.2	50.7	-	<0.001	37.3	42.3	<0.001
	Dose 2	33.8	80.6	46.3	<0.001	20.2	-	-
	p-value^2^	<0.001	<0.001	-		<0.001	-	
Any systemic reaction								
	Dose 1	13.7	48.8	-	<0.001	41.1	49.4	<0.001
	Dose 2	35.7	82.1	46.4	<0.001	23.3	-	-
	p-value^2^	<0.001	<0.001	-		<0.001	-	
Limits on daily life activities								
	Dose 1	1.7	21.5	-	<0.001	19.0	26.0	<0.001
	Dose 2	13.8	64.8	21.5	<0.001	3.8	-	-
	p-value^2^	<0.001	<0.001	-		<0.001	-	

mRNA, messenger RNA; ChAd, AstraZeneca COVID-19 vaccine (ChAdOx1-S); BNT, Pfizer-BioNTech COVID-19 vaccine (BNT162b2); Ad26, Janssen COVID-19 vaccine (Ad26.COV2.S); mRNA-1273, Moderna COVID-19 vaccine (messenger RNA-1273).

1 Received ChAd for the first dose and BNT for the second dose.

2 Calculated by the chi-square test.

**Table 3.** Factors associated with any adverse events and health status after coronavirus disease 2019 (COVID-19) vaccination^1^

Variables		Dose 1^*^			Dose 2^*^		
		Any local reaction	Any systemic reaction	Limitations on daily activities	Any local reaction	Any systemic reaction	Limitations on daily activities
Female (reference: male)		1.66 (1.56, 1.77)	1.66 (1.56, 1.76)	1.67 (1.53, 1.81)	1.96 (1.84, 2.09)	1.92 (1.81, 2.04)	1.92 (1.77, 2.09)
Age (yr) (reference: ≥60)							
	≤29	3.08 (2.68, 3.54)	2.95 (2.58, 3.37)	3.95 (3.15, 4.94)	1.97 (1.78, 2.18)	2.01 (1.86, 2.21)	1.93 (1.67, 2.22)
	30-39	2.92 (2.55, 3.36)	2.87 (2.52, 3.28)	3.51 (2.81, 4.38)	2.24 (2.05, 2.46)	2.24 (2.04, 2.45)	2.28 (2.02, 2.58)
	40-49	1.97 (1.71, 2.26)	1.96 (1.72, 2.25)	2.19 (1.74, 2.74)	1.68 (1.54, 1.84)	1.74 (1.60, 1.90)	1.65 (1.47, 1.86)
	50-59	1.39 (1.20, 1.61)	1.44 (1.26, 1.66)	1.47 (1.16, 1.85)	1.32 (1.21, 1.44)	1.34 (1.23, 1.46)	1.30 (1.15, 1.46)
Brand (reference: BNT)							
	mRNA-1273	5.73 (5.28, 6.22)	4.76 (4.40, 5.16)	11.75 (9.95, 13.86)	7.35 (6.71, 8.06)	7.54 (6.87, 8.27)	10.64 (9.63, 11.76)
	ChAd	4.76 (4.43, 5.11)	4.81 (4.49, 5.15)	15.54 (13.27, 18.20)	0.49 (0.46, 0.53)	0.54 (0.51, 0.58)	0.25 (0.22, 0.29)
Heterologous (ChAd/BNT)					2.04 (1.91, 2.19)	1.91 (1.78, 2.04)	2.08 (1.90, 2.28)
Ad26		6.57 (6.00, 7.20)	7.67 (7.03, 8.38)	26.25 (22.03, 31.27)			

Values are presented as odds ratio (95% confidence interval). ChAd, AstraZeneca COVID-19 vaccine (ChAdOx1-S); BNT, Pfizer-BioNTech COVID-19 vaccine (BNT162b2); Ad26, Janssen COVID-19 vaccine (Ad26.COV2.S); mRNA-1273, Moderna COVID-19 vaccine (messenger RNA-1273).

1 For the brand analysis in the dose 2 section, the reference group is BNT/BNT, “mRNA-1273” refers to mRNA-1273/mRNA-1273, and “ChAd” refers to ChAd/ChAd.

* p<0.05.
